# Development and validation of a HPLC/FLD method combined with online derivatization for the simple and simultaneous determination of trace amino acids and alkyl amines in continental and marine aerosols

**DOI:** 10.1371/journal.pone.0206488

**Published:** 2018-11-12

**Authors:** Xiongfeng Huang, Shuh-Ji Kao, Jing Lin, Xiaofei Qin, Congrui Deng

**Affiliations:** 1 Shanghai Key Laboratory of Atmospheric Particle Pollution and Prevention (LAP), Department of Environmental Science and Engineering, Fudan University, Shanghai, China; 2 State Key Laboratory of Marine Environmental Sciences, Xiamen University, Xiamen, China; 3 Fuzhou Research Academy of Environmental Science, Fuzhou, China; Indian Institute of Chemical Technology, INDIA

## Abstract

A method was developed for simultaneous determination of 15 amino acids and 7 alkyl amines. The method was based on the employment of high performance liquid chromatography/fluorescence detection and online derivatization with *o*-phthaldiadehyde. The 22 derivatives were separated within 30 min including the equilibration time and detected by a fluorescence detector at an excitation wavelength of 230 nm and emission wavelength of 450 nm. The analysis procedure was satisfactorily validated by the reproducibility, recovery, linearity and detection limit of the analytes. The relative standard deviations (RSDs) of retention time and peak area for individual amino acids and alkyl amines were consistently less than 0.30% and 2.35%, respectively. Good recovery values ranging from 70% to 109% were obtained. The proposed method showed good linearity (R^2^≥0.99) in the range of 0.125–125 μM/L for amino acids and 2.5–5000 ng/L for alkyl amines. The detection limit ranged from 0.13 pM to 0.37 pM for individual amino acids and from 0.9 ng to 7.2 ng for individual alkyl amines. The developed and validated method was successfully applied to the quantitative analysis of amino acids and alkyl amines in continental and marine aerosols in China. Among the identified organic nitrogen compounds, 7 amino acids and 6 alkyl amines were detected in every aerosol sample. Glycine was the dominant amino acid, with the average of 130.93 pmol/m^3^ (accounting for 83% of the total amino acids) and 137.22 pmol/m^3^ (accounting for 66% of the total amino acids) in continental and marine aerosols in China, respectively. Methylamine and ethanolamine were the most abundant alkyl amines, contributing 87% and 64% to the total alkyl amines in continental and marine aerosols in China, respectively. This work provided an accurate, sensitive and simple method to determine simultaneously amino acids and alkyl amines, and applied the proposed method to the first investigation of amino acids in Shanghai and amino acids and alkyl amines in Huaniao Island in China. The finding of considerable amino acids and alkyl amines in continental and marine aerosols may exert significant implications on nitrogen cycling and atmospheric chemistry.

## Introduction

Nitrogen controls both the diversities and quantity of living organisms in marine and terrestrial environments on Earth. The atmosphere can exert a substantial impact on the transport and transformation of nitrogen worldwide [1]. Increasing studies have suggested that organic nitrogen represents a ubiquitous and significant component of atmospheric nitrogen [2,3]. The contribution of organic nitrogen to the total nitrogen varied from site to site but the percentage was consistently around one third of the total nitrogen [2,4,5].

Considerable amounts of organic nitrogen have been found in different regions. For example, Miyazaki *et al*.(2014) [4] detected considerable atmospheric organic nitrogen in a deciduous broadleaf forest in northern Japan; Wang *et al*.(2010) [6] and Huang *et al*.(2012) [7] identified numerous nitrogen-containing organics in Shanghai. To appropriately characterize their environmental effects, knowledge of the chemical forms of organic nitrogen is required. A variety of organic nitrogen compounds have been detected or hypothesized to exist in the atmosphere. Of these compounds, amino acids and amines have probably been identified most extensively recently attributed to the following reasons [8,9]. First, as water-soluble organic carbon, amino acids and amines exerts the potential effect on the hygroscopic growth of particles and even affects visibility [10–13]. Second, considering the presence of basic nitrogen functional groups, amino acids and amines may contribute to the buffering capacity of particles [14,15]. Finally, amines present a potential toxic risk to human health because they are precursors of carcinogenic compounds such as nitroamines and nitramines [16]. Taken together, amino acids and amines appears to represent a considerable portion of atmospheric nitrogen and therefore might have significant effects on the environment and human health. Facchini *et al*. (2008) [17] and Müeller *et al*. (2009) [18] measured significant amines in North Atlantic and Cape Verde, respectively. Zhang and Anastasio (2003) [14] found considerable amounts of free and combined amino acids in PM_2.5_ and fog waters from Northern California, and Mace *et al*. (2003) [19–21] detected significant concentrations of amino compounds in Amazon Basin aerosols [19], Cape Grim rain and aerosols [20], as well as in eastern Mediterranean rain and aerosols [21]. Finessi *et al*. (2012) [22] and Dall’Osto M. *et al*. (2017) [23] found significant amines in aerosols in Forest and polar regions.

Both amino acids and amines are important and ubiquitous components of organic nitrogen in the atmosphere. However, most studies of organic nitrogen compounds in the atmosphere have focused on only amino acids or amines in individual region, and there are rare published reports of simultaneous measurements of amino acids and amines in both continental and marine atmosphere. This is largely attributed to the fact that few analytical techniques are sufficiently effective to determine atmospheric amino acids and amines simultaneously. Although numerous measurement methods for amino acids have been reported, the effective separation and detection remains extremely challenging because of their high polarity and lack of strong chromospheres [24, 25]. Quantitative analysis of amines is also difficult due to the similar limitations [26–29]. Therefore, studies on the simultaneous determination of atmospheric amino acids and alkyl amines are very rare. Even in the simultaneous determination of amino acids and amines, several disadvantages of the reported methods have been identified including co-elution of analytes, a limited number of organic nitrogen compounds, laborious derivatization procedures, long analysis time, matrix interferences and so on[24,30].

Increasing evidence has led to the recognition that there is a need for the simultaneous quantification of amino acids and alkyl amines at various concentrations presenting in the atmospheric matrix. The most common measurement methods for amino acids and alkyl amines include gas chromatography (GC) [25,30] and high performance liquid chromatography (HPLC) [24,30]. As amino acids and amines are basic and polar compounds, their separation and peak-shape performance on GC columns are often complicated for GC stationary-phase choices. Derivatization can improve their GC separation and peak shape, but complete separation is seldom achieved. Another disadvantage of GC is time-consuming and laborious derivatization. Several HPLC analysis techniques have also been explored. Prior to analysis, amino acids and amines must also be derivatized. Proper selection of a chromophoric derivatization reagent for both amino acids and amines is an important consideration in HPLC analysis. The most commonly-used derivatization reagents include 7-fluoro-4-nitrobenzo-2-oxa-1,3-diazole (NBD-F), 4-dimethylaminoazobenzenesulfonylchlorode (Dabsyl-Cl), 9-fluorenylmethylchloroformate (FMOC), 6-aminoquinolyl-*N*-hydroxy-succinimidyl carbamate (AQC), and o-phthaldiadehyde (OPA) [24, 31–35]. The derivatization of NBD-F, Dabsyl-Cl and FMOC-Cl all presents the interference effect of reagents unless the reagents are extracted prior to chromatographic analysis or the molar excess of reagents is carefully limited. Although the unique fluorescence properties of AQC-related compounds permit the analysis of derivatized samples without prior reagent removal yet with minimal reagent interference, the cost is relatively high and the derivatization procedure requires manual operation [30]. To the best of our knowledge, OPA can simultaneously react with amino acids and amines under fairly mild conditions to form highly fluorescent substances and the LC sampler allows auto-mixing between derivatizing reagents and analytes. In this study, we utilized OPA online derivatization in order to reduce labor, improve accuracy and save time. Considering the advantages of HPLC autosampler, the high sensitivity of fluorescence detection (FLD) and the mild conditions of OPA derivatization, HPLC/FLD combined with online OPA derivatization was employed to separate and detect amino acids and alkyl amines in this study.

The goal of this study is to provide an effective measurement method for the simultaneous quantification of amino acids and alkyl amines in aerosols without laborious operation and excess time-consumption. As far as we are aware, very limited information is available on the atmospheric amino acids and alkyl amines in China. Therefore, another goal of this study is to apply the proposed method to the quantitative analysis and provide an investigation of amino acids and alkyl amines in both continental and marine aerosols in China.

## Material and methods

### Materials and reagents

Acetonitrile (ACN) and methanol (MeOH) of HPLC grade were obtained from Fisher Chemical. 3-Mercapto-propionic acid (C_3_H_6_O_2_S, purity≥99%), OPA (C_8_H_6_O_2_, purity≥99%) and boric acid (H_3_BO_3_, purity≥99.5%) were obtained from Sigma-Aldrich. Phosphoric acid (H_3_PO_4_, purity≥85.0%), sodium hydroxide (NaOH, purity≥98%), potassium dihydrogen phosphate (KH_2_PO_4_, purity ≥99.5%), and sodium tetraborate decahydrate (Na_2_B_4_O_7_∙10H_2_O, purity≥99.5%) were purchased from SCRC (China). Ultrapure water was freshly obtained from a Milli-Q Plus system (≥18.2MΩ cm, Millipore). All chemicals were of reagent grade or better.

Methylamine, ethylamine, propylamine, butylamine, pentylamine (purity≥99%), hexylamine and ethanolamine, which were used for standard preparation, were all purchased from Aldrich. The analyzed amino acids were as follows: alanine (Ala), arginine (Arg), aspartic acid (Asp), histidine (His), glutamic acid (Glu), glycine (Gly), isoleucine (Ile), leucine (Leu), methionine (Met), phenylalanine (Phe), serine (Ser), threonine (Thr), tyrosine (Tyr), valine (Val), and lysine (Lys). The concentration of the amino acid standard solution, which was received from Sigma-Aldrich, was 2.5 μmol/mL dissolved in 0.1N HCl solution.

### Online derivatization

The derivatization reaction of amino acids and alkyl amines with OPA was performed online in the HPLC autosampler. Briefly, the derivatization reaction was performed by sufficiently mixing of borate buffer (0.1 M Na_2_B_4_O_7_∙10H_2_O buffer at pH 10.2), derivatization reagent (prepared as 5 mM OPA, 225 mM 3-mercapto-propionic acid in 0.1 M borate buffer), injection diluent (1 M acetic acid) and aerosol samples at room temperature. After derivatization, the mixture was injected into the column. The entire process took ~5 min, including needle washing. It is noteworthy that the derivatizing reagents should be frozen and kept in the dark until use, and the borate buffer should be freshly prepared.

### Instrumental analysis

Briefly, a Thermo Dionex high performance liquid chromatography system equipped with a DGP pump, a vacuum degasser, an autosampler with a thermostat, a thermostatted column compartment and a fluorescence detector (λ_ex_ = 230 nm and λ_em_ = 450 nm) was utilized to quantify the highly fluorescent sulfonatoisoindole derivatives of 15 amino acids and 7 alkyl amines in aerosols. A Hypersil Gold column (ThermoScientific, 5 μm, 4.6×150 mm) coupled to a C18 guard column (ThermoScientific, 5 μm, 4.6×10 mm) provided the means for separating the derivatized amino acids and alkyl amines at a flow rate of 1.5 mL/min (column temperature of 35°C). The derivatives were eluted with a gradient program by two eluents: eluent A (50 mM KH_2_PO_4_ buffer, pH4.6) and eluent B (ACN: MeOH: H_2_O, 45:45:10). The chromatographic elution gradients were as follows: maintaining 28% B for 3 min, 28–80% B for 10 min, holding at 80% B for 5 min, equilibration with 28% B before returning to 28% B for 0.5 min.

### Sample collection and processing

PM_2.5_ samples were simultaneously collected in Shanghai (n = 11), and Huaniao Island (n = 11), from January 8, 2013 to January 19, 2013. Particulate matters were collected on pre-combusted (6 h at 450°C in a muffle furnace) quartz microfiber filters (Whatman Company, UK). Sampling intervals were generally taken as 24 h. Blank samples were collected by loading, carrying and installing the filter holder with the pump closed. The collected filters were stored at -20°C in pre-combusted aluminum foil envelops, placed in separate plastic bags. Samples were ultrasonically extracted in methanol. After concentration and filtration, the extract was directly analyzed. Extreme care was exercised to eliminate contamination.

## Results and discussion

### Quality control

Each derivative was evaluated in terms of the reproducibility of retention time, peak area, and recovery studies. The relative standard deviations (RSDs) of retention time for individual amino acids and alkyl amines were consistently below 0.30%, and the RSDs of peak area were consistently less than 2.0%, except 2.35% for lysine and 2.11% for hexylamine. Recovery was estimated through analysis by adding various known amounts of each amino acid and alkyl amine to quartz filters, and then performing pretreatment as aerosol samples. Good recovery values were obtained with the mean recoveries ranging from 70% to 109%. Blanks were also analyzed and all analytes were below the detection limit.

Method sensitivity was evaluated by measuring the detection limit, which was defined as the concentration with a signal-to-noise ratio (determined by peak area) of at least 3 (n = 12). The detection limit ranged from 0.13 pM to 0.37 pM for individual amino acids and from 0.9 ng to 7.2 ng for individual alkyl amines. The content units of the two organic nitrogen compounds were different originally in the standard solutions purchased, with mM/L for amino acids and mg/L for alkyl amines.

To verify the linearity of the response of different derivatives, standard solutions of 15 amino acids and 7 alkyl amines in the concentrations from 0.125 μM/L to 125 μM/L and from 2.5 μg/L to 5000 μg/L, respectively, were prepared and analyzed in triplicate. Linearity was evaluated by the determination coefficient of the least square regression (*R*^2^). The coefficient of regression exceeded 0.995 for all standard calibration curves, except for pentylamine, which showed a coefficient of regression of 0.9947. The results indicated that amino acids and alkyl amines showed good linearity spanning over three orders of magnitude under the proposed conditions.

Reproducibility, recovery, detection limit and linearity were assayed with satisfactory results (Table 1). All of these results suggested that the proposed method was reproducible, sensitive, quantitative, and linear over a wide dynamic range.

**Table 1:** Reproducibility, detection limit, and calibration curve of each analyte.

Compound	RSD of RT (%)	RSD of A (%)	DL	*R*^2^
**Asp**	0.30	1.55	0.31 pM	1.00
**Glu**	0.21	1.46	0.37 pM	0.9994
**Ser**	0.11	1.57	0.31 pM	0.9996
**His**	0.06	1.82	0.30 pM	0.9993
**Gly**	0.12	1.06	0.33 pM	0.9998
**Thr**	0.08	1.50	0.31 pM	0.9993
**Arg**	0.07	1.78	0.13 pM	0.9994
**Ala**	0.10	1.47	0.31 pM	0.9996
**Tyr**	0.11	1.83	0.13 pM	0.9993
**Val**	0.10	1.62	0.29 pM	0.9991
**Met**	0.12	1.78	0.33 pM	0.9995
**Phe**	0.06	1.41	0.29 pM	0.9987
**Ile**	0.06	1.70	0.27 pM	0.9978
**Leu**	0.04	1.99	0.31 pM	0.9977
**Lys**	0.05	2.35	0.38 pM	0.9976
**Methylamine**	0.05	1.83	3.9 ng	0.9989
**Ethylamine**	0.05	1.11	1.5 ng	0.9959
**Propylamine**	0.03	1.54	7.2 ng	0.9975
**Butylamine**	0.03	1.38	1.8 ng	0.9947
**Pentylamine**	0.03	1.54	2.8 ng	0.9975
**Hexylamine**	0.03	2.11	6.8 ng	0.9976
**Ethanolamine**	0.10	1.47	0.9 ng	0.9997

DL: detection limit; RT: retention time; A: area.

### Overall evaluation of the proposed method

Given the fact that both amino acids and alkyl amines contain the–NH_2_ functional group, amino acids and alkyl amines can simultaneously derivatize with OPA. Additionally, the mild conditions of the derivatization reaction and the automixing function of the HPLC autosampler provide potential for online derivatization. Finally, fluorescence detection and experimental conditions offer sufficient sensitivity, selectivity, and linearity for quantifying variable levels of amino acid and alkyl amine in the atmospheric matrix. Therefore, the proposed method combined advantages of simultaneous, simple and sensitive determination of amino acids and alkyl amines in complicated matrices in a short time. Another potential highlight of the proposed method is the simultaneous determination of amino acids, amines and other NH_2_-containing compounds.

**Fig 1:**
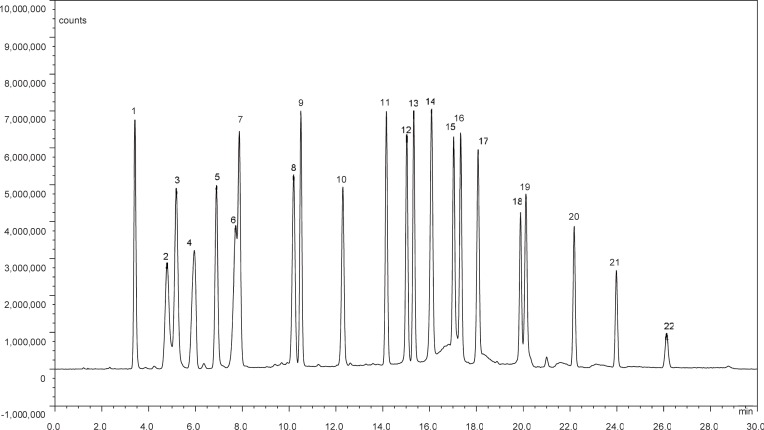
Chromatogram of 15 amino acid (1.25 μM/L) and 7 alkyl amine (50 μg/L) standard solution. (1) His (2) Asp, (3) Ser, (4) Arg, (5) Glu, (6) Gly, (7) Thr, (8) Ala, (9) Tyr, (10) ethanolamine, (11) Met, (12) Val, (13) Phe, (14) methylamine, (15) Ile, (16) Leu, (17) ethylamine, (18) Lys, (19) propylamine, (20) butylamine, (21) pentylamine, and (22) hexylamine.

The results mentioned above showed that the proposed method was capable of separating 15 amino acids and 7 alkyl amines within 30 min including equilibration time, as shown in Fig 1. The retention time of the 15 amino acids and 7 alkyl amines ranged from 3.4 min to 26.2 min. Twenty-two analytes were separated completely, except for Gly and Thr (neither complete separation nor co-elution). Compared with the reported method in Table 2, the proposed method offered the advantages of low analysis time, easy derivatization operation, good separation resolution, and high method accuracy.

**Table 2:** Summary of analysis methods of amino acids and amines by HPLC.

Matrix/ Derivatization	Resolution	RSD	Compounds/min	Reference
**Atmospheric, OPA offline**	Separate partly	9.4%	20 AA, 2 A /39 min	[14]
**Musts/Wines, OPA+FMOC**	—	0.5–19.02%	32 AA/138 min	[36]
**AQC, offline**	—	—	21 A, 3 A/—	[32]
**Atmosphere, OPA offline**	Separate completely	—	4 A/8 min	[33]
**Atmosphere, OPA online**	Separate completely	0.02–1.52%	8A/13 min	[35]
**Atmosphere, OPA online**	Separate basically	1.06–2.35%	15 AA, 7 A/30min	This study

“—” denoted not reported. “Separately completely” denoted that the resolution of all analytes was greater than or equal to 1.5. “Separately basically” denoted that the resolution of most analytes was greater than or equal to 1.5, apart from that of a few analytes which was separated but their resolution was less than 1.5 (such as the resolution between Gly and Thr in Fig 1 in this study). “Separate partly” denoted co-elution of some analytes, such as Gly and Thr in reference 14. “AA” and “A” denoted amino acids and amines, respectively. RPD: relative percent difference.

### Atmospheric application

**Fig 2:**
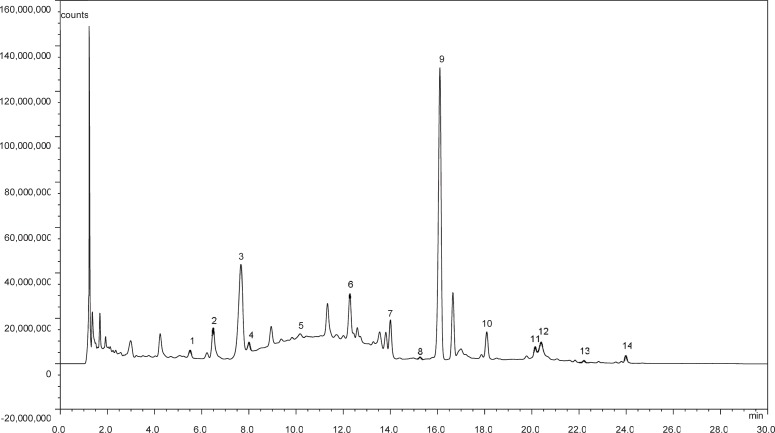
Chromatogram of an aerosol sample. (1) Ser, (2) Arg, (3) Gly, (4) Thr, (5) Ala, (6) ethanolamine, (7) Met, (8) Phe, (9) methylamine, (10) ethylamine, (11) Lys, (12) propylamine, (13) butylamine, (14) pentylamine.

The proposed method was successfully applied in quantitative analysis of 15 amino acids and 7 alkyl amines in the continental and marine aerosols, as shown in Fig 2. During our observations, 12 amino acids (including Asp, Ser, Gly, Thr, Arg, Ala, Val, Met, Phe, Ile, Leu, and Lys) and 6 alkyl amines (including ethanolamine, methylamine, ethylamine, propylamine, butylamine, and pentylamine) were identified and quantified in PM_2.5_ over Shanghai, whereas 11 amino acids (including Glu, Ser, Gly, Thr, Ala, Val, Met, Phe, Ile, Leu, and Lys) and 6 alkyl amine (including ethanolamine, methylamine, ethylamine, propylamine, butylamine, and pentylamine) were found over Huaniao Island. Among the detected organic nitrogen compounds, 7 amino acids and 6 alkyl amines were identified to be present in every sample from Shanghai, whereas 11 amino acids and 6 alkyl amines in every sample from Huaniao Island. Moreover, 7 amino acids and 6 alkyl amines were both detected in every sample over both Shanghai and Huaniao Island. The concentrations of individual amino acids and alkyl amines detected are listed in Table 3.

**Table 3:** Concentrations of amino acids (in pmol/m^3^) and alkyl amines (in ng/m^3^) in continental and marine aerosols.

Compound	Average Concentration		Concentration Range	
	Shanghai	Huaniao Island	Shanghai	Huaniao Island
**Asp**	2.76	ND	ND-6.15	ND
**Glu**	ND	9.72	ND	8.04–11.05
**Ser**	3.39	1.70	1.32–7.69	1.18–3.26
**His**	ND	ND	ND	ND
**Gly**	130.93	137.22	67.45–251.52	20.34–275.07
**Thr**	9.06	6.32	2.13–44.37	3.72–11.06
**Arg**	1.07	ND	ND-3.98	ND
**Ala**	4.06	3.42	1.06–8.99	1.74–6.35
**Tyr**	ND	ND	ND	ND
**Val**	0.63	0.91	0.09–1.54	0.31–1.36
**Met**	1.90	7.22	0.44–3.74	1.75–13.06
**Phe**	0.71	1.21	ND-1.54	0.44–2.31
**Ile**	0.48	25.77	ND-2.48	7.69–43.99
**Leu**	0.17	6.27	ND-0.40	1.56–13.01
**Lys**	1.89	9.78	0.49–3.98	3.03–25.62
**Sum of AA**	157.05	209.54	79.20–295.96	118.66–328.11
**Methylamine**	2.34	4.76	1.00–5.21	0.27–7.04
**Ethylamine**	0.24	0.82	0.09–0.37	0.37–1.78
**Propylamine**	0.09	2.47	0.01–0.38	0.76–4.03
**Butylamine**	0.22	0.04	0.02–1.35	ND-0.15
**Pentylamine**	0.06	0.24	0.01–0.13	0.07–0.40
**Hexylamine**	ND	ND	ND	ND
**Ethanolamine**	1.80	1.49	0.26–4.21	0.84–5.62
**Sum of A**	4.75	9.82	2.21–9.48	6.25–13.30

ND: not detectable. AA and A denoted amino acids and alkyl amines, respectively.

From Table 3, the level of and between different amino acids fluctuated widely, often with over one order of magnitude. For example, the concentrations of Thr in Shanghai ranged from 2.13 pmol/m^3^ to 44.37 pmol/m^3^, and the average concentration of Gly (130.93 pmol/m^3^) was over two orders of magnitude higher than that of Phe (0.71 pmol/m^3^) in Shanghai. In our measurements, Gly was the most abundant amino acid among the amino acid species both in Shanghai and Huaniao Island. This fact might be attributed to the low reactivity of glycine [37]. The concentrations of Gly in PM_2.5_ were in the range of 67.45–251.52 pmol/m^3^ averaging 130.93 pmol/m^3^ over Shanghai, and 20.34–275.07 pmol/m^3^ averaging 137.22 pmol/m^3^ over Huaniao Island. Methylamine was the dominant alkyl amine, followed by ethanolamine, in both Shanghai and Huaniao Island. Levels of methylamine in PM_2.5_ ranged from 1.00 ng/m^3^ to 5.21 ng/m^3^ with the average of 2.34 ng/m^3^ over Shanghai and from 0.27 ng/m^3^ to 7.04 ng/m^3^ with the average of 4.76 ng/m^3^ over Huaniao Island. Considerable amino acids and alkyl amines have been detected in particulate matters at various locations. For example, amino acids were found in PM_2.5_ in California [14,38] and Venice [39]; alkyl amines were identified in Eastern Mediterranean [40], North Atlantic Ocean [17] and other environments such as boreal forest and polar regions [41, 42]. This study is consistent with previous studies, which also detected a marked predominance of Gly in aerosols in Venice [39]. Furthermore, a substantial contribution of Gly was observed in PM_2.5_ in California, but in their measurements Ornithine was the most abundant free amino acid [14]. Comparison of the total average concentrations of free amino acids with previously published data showed similar concentrations to those obtained by Zhang and Anastasio [14] (184 pmol/m^3^), lower concentrations to those obtained by Barbaro et al [39] (334 pmol/m^3^). Based on our available data, methylamine and ethanolamine appear to be ubiquitous and significant in the atmospheric environment. This is in agreement to the measurements of amines in continental and marine aerosols [14, 17, 40].

**Fig 3:**
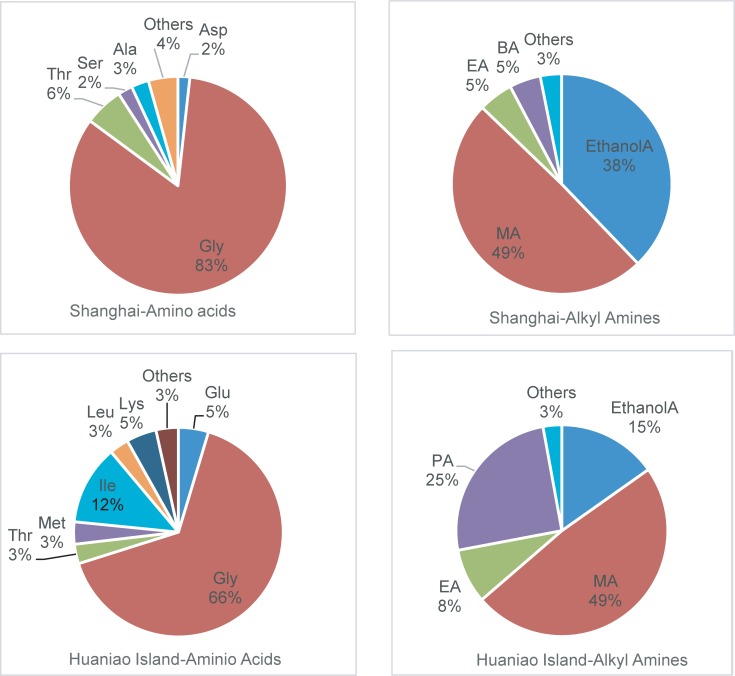
Average contributions of individual amino acids to the amino acid pool (in pmol/m^3^) and individual alkyl amines to the alkyl amine pool (in ng/m^3^) in PM_2.5_ over Shanghai and Huaniao Island, respectively. MA, EA, PA, BA, EthanolA denoted methylamine, ethylamine, propylamine, butylamine, and ethanolamine, respectively. The category “others” included other detectable analytes.

The average contributions for individual amino acids to the amino acid pool are also presented in Fig 3 (n = 11 for Shanghai as well as for Huaniao Island). On average, Gly alone accounted for 83% of the total amino acids in Shanghai and 66% on Huaniao Island. The total of methylamine and ethanolamine contributed over 60% to the total alkyl amines in both sampling sites.

**Fig 4:**
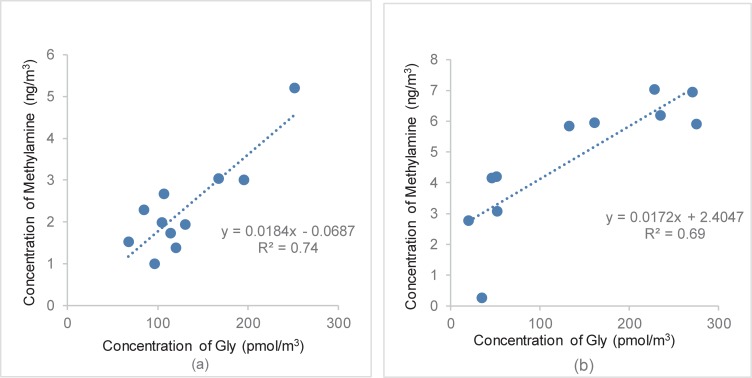
Correlation between Gly and methylamine in PM_2.5_ over (a) Shanghai and (b) Huaniao Island.

Also, the correlation (R^2^ = 0.74, n = 11 for Shanghai, and R^2^ = 0.69, n = 11 for Huaniao Island) between Gly and methylamine was examined, as shown in Fig 4. It was found that the level of Gly and methylamine correlated well in their respective sampling sites, thereby suggesting that they likely originated from the same sources. A surprising finding was the presence of considerable amounts of Met over Huaniao Island, which was not generally detected in environmental samples. Its presence might reflect that a proportion of the amino acids on Huaniao Island were formed from protein decomposition or through gas phase reactions [19].

## Conclusion

A simple, sensitive method for determining 15 amino acids and 7 alkyl amines in the atmosphere was developed and described in detail in this study. The developed method offered the advantages of quantifying amino acids and alkyl amines simultaneously and sensitively in a short time (≤30 min). An additional advantage was the employment of OPA online derivatization, which reduced time consumption and improved detection accuracy. The ability to more reliably quantify amino acids and alkyl amines is beneficial to further research.

Moreover, the proposed method was successfully applied to the simultaneous determination of amino acids and alkyl amines in continental and marine aerosols in China. Considerable species and amounts of amino acids and alkyl amines were detected. This study also provided the investigation of amino acids and alkyl amines in continental and marine aerosols in China.
